# Assessing regional competitiveness in Peru: An approach using nonlinear machine learning models

**DOI:** 10.1371/journal.pone.0318813

**Published:** 2025-02-25

**Authors:** Yvan J. Garcia-Lopez, Luis A. del Carpio Castro

**Affiliations:** 1 CENTRUM Católica Graduate Business School (CCGBS), Lima, Peru; 2 Pontificia Universidad Católica del Perú (PUCP), Lima, Peru; Czestochowa University of Technology: Politechnika Czestochowska, POLAND

## Abstract

This study addresses the challenges of measuring regional competitiveness using traditional methods, due to the inherent complexity and non-linearity of its determinants’. The development of new Machine Learning (ML) models allows the creation of predictive models capable of handling this type of data, providing actionable insights. The objective of the study was to develop and test the use of non-linear Machine Learning models to measure the regional competitiveness in Peru, at the sub-national level. The research uses the ODD (Overview, Design Concepts, and Details) protocol to ensure a transparent and replicable methodology. The impact of ML on the Peruvian Regional Competitiveness Index (IRCI) is examined across 25 regions from 2016 to 2023, focusing on five key pillars: economy, government, infrastructure, businesses, and people. A suitability index (IoI) was developed to assess how well the pillar components align with ML. Data provided by CENTRUM PUCP was subjected to exploratory data analysis (EDA) to address variability among pillar scores and their effects on competitiveness. Six nonlinear machine learning models (Gradient Boosting, Random Forest, XGBoost, AdaBoost, Neural Networks, and Decision Trees) were applied, and the machine learning models with the highest predictive accuracy were Gradient Boosting and Random Forest. Performance metrics include MSE values of 1.1399 and 1.3469, RMSE values of 1.0677 and 1.1606, and R^2^ values of 0.9768 and 0.9729, respectively. These results demonstrate the effectiveness of machine learning in analyzing the complexity of regional competitiveness data, identifying influential variables, and reducing score distortions. The findings provide a data-driven framework for policymakers to improve regional competitiveness, which promotes academic knowledge and practical applications for sustainable development.

## Introduction

Regional competitiveness is an important indicator of the economic and social development of any country, particularly due to its relationship with economic development and improvement in the quality of life of the population [1,2]. Measuring this competitiveness in the Peruvian context becomes a fundamental challenge to designing effective policies that promote balanced and sustainable growth among the various regions.

It has been observed that traditional measurement methods often fail to capture the complexity and dynamism of regional factors that compose the dimensions of regional competitiveness and which in many cases are interrelated [3]. Therefore, applying non-linear machine learning (ML) models offers a promising alternative due to its ability to handle and analyze large volumes of data with multiple interdependent variables. This research focuses on applying these advanced machine learning models to evaluate the Peruvian Regional Competitiveness Index (PRCI) and discover significant patterns and correlations in the data of the 25 regions of Peru between 2016 and 2023.

The COVID-19 pandemic has forced many companies to accelerate their digital transformation strategies to continue meeting the changing needs of their customers [4]. Still, the complexity of the transformation process affects the emergence of challenges and problems that must be overcome to create innovative digital models that enable the use of the full potential in the organization [5]. This has led to significant growth in the global software market, as the COVID-19 pandemic in 2020 forced Agile Software Development Teams (ASDT) to quickly transition to remote work and adapt to new business circumstances [6].

On the other hand, Machine Learning emerges as a subfield of artificial intelligence that allows computers to learn about something they have not been explicitly programmed to do [7]. Many researchers have developed various intelligent techniques, such as deep learning (DL) and machine learning (ML), which can help drive research in the field of competitiveness [8]. Competition drives progress, but a company must be highly competitive to function and develop optimally [9].

The definition of regional competitiveness is not unique in academic literature and is the subject of debate among various authors, who consider it to be a developing concept [10–15]. According to the Oxford Dictionary, competitiveness is the ability of an economy to meet “growing aggregate demand and sustain exports” [16]. In turn, competitiveness at the microeconomic level is the ability of an organization to compete successfully with its business rivals. Aiginger proposed to define “competitiveness” as “the ability of a country or location to create well-being” [17].

Michael Porter, one of the significant contributors to modern competitiveness theory, noted that competitiveness can be represented in several layers: the resources available to the country, the intermediate layer, which is represented by macroeconomic competitiveness, and the microeconomic layer as a combination of the environment surrounding the firm itself and clusters [18]. Firms with higher levels of regional competitiveness are associated with higher levels of well-being. Thus, for a given region, competitiveness must increase the market share of a particular industry and the population’s standard of living [19].

The Organization for Economic Cooperation and Development [20], defines regional competitiveness as the ability to attract and retain enterprises that improve or maintain the standard of living of the population, as well as their ability to improve the GPD. This definition takes into account the importance of organisations in regional development and their impact on the well-being of the population [21,22]. In addition, Kitson et al. [23] mention that regional competitiveness is the ability of regions or cities to compete with each other, which is key for decision-making when implementing public policies aimed at stimulating economic development.

These contributions have been important for the construction of the concept of regional competitiveness, highlighting the importance between economic growth, increased wealth, productivity and general welfare [24–28]. In this opportunity, and based on the literature review, the definition of regional competitiveness will be the one mentioned by Del Carpio et al. [29], which integrates these contributions and understands regional competitiveness in three different ways: competition between territorial spaces for investment or public resources; the search for economic development through the promotion of business productivity; and finally, the management of resources through the design of public policies aimed at improving the welfare of its citizens and generating an appropriate environment for investment.

**Fig 1:**
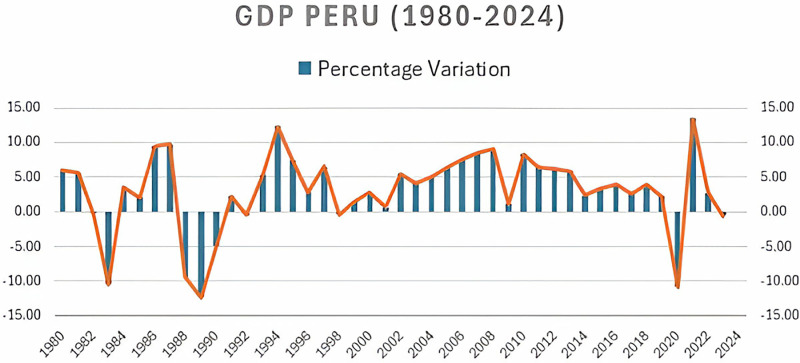
GDP Percentage Change 1980–2024. Data obtained from BCRP (https://estadisticas.bcrp.gob.pe/estadisticas/series/anuales/resultados/PM04863AA/html).

Since the 1960s, Peru has tried, very slowly, to emulate policies other Latin American countries applied to promote internal industrialization, reducing dependence on the export of raw materials and contributing to diversifying the production structure [30]. Although the application of these policies had ups and downs, it was not until the 1990s—under the election of President Alberto Fujimori (1990-2000) – that the country radically departed from this model and undertook a “neoliberal revolution” [31]. The exhaustion of the import substitution model became evident long before the beginning of the 1990s. Still, the “critical juncture” of the late 1980s created the economic and political conditions that facilitated a radical change in the direction of Peruvian policies [29] (Fig 1).

Given the above, between 2003 and 2013, the Peruvian economy went through an unusual period of expansion in terms of historical trends and growth patterns in other parts of Latin America. The period became known as “the Peruvian miracle.” Not only did production expand in all economic sectors, but Peru’s exports experienced a sudden increase.

The period saw rising real incomes and substantial reductions in poverty rates until 2008. However, the employment structure remained such that the country had one of the region’s most significant percentages of the workforce in the informal sector. Still, the growth bonanza had come to an end [31]. The validity of the growth model by his more democratic presidential successors had increasingly become the subject of question and controversy.

During the period of rapid growth, business confidence remained solid, and critical decision-making positions were held by those most committed to the model. To address these issues [29], governments often resort to composite indicators, such as those proposed by the OECD [32] or the Global Competitiveness Index (GCI) developed by the World Economic Forum [33], based on Porter [34] to measure competitiveness. While these indices are widely used to measure the competitiveness of countries [35–38], at the regional level, there is no index comparable to the GCI due to the lack of consensus on the definition of regional competitiveness and the scarcity of comparable data [39]. However, there are local indices with methodologies similar to the GCI that assess competitiveness in specific regions of countries. These were used to rank Peruvian regions according to their implied levels of competitiveness for the year 2011.

From 2015 to 2019, the limitations of the neoliberal model became increasingly evident. This occurred as the growth pattern slowed, many did not receive benefits, and public confidence in the business class was undermined by corruption scandals [40,41].

Between 2020 and 2023, the Peru Regional Competitiveness Index (PRCI) experienced significant changes due to various factors that affected the region’s development. The PRCI measures the capacity of the regions to generate well-being and sustainable development based on indicators in areas such as infrastructure, health, education, innovation, and economy. Some of the most relevant aspects of the index’s performance in this period include:

### Impact of the Pandemic (2020–2021)

The COVID-19 pandemic exacerbated regional disparities in Peru, affecting less competitive regions due to their lack of adequate health infrastructure and limited response capacity in health, education, and economic systems. With better health services, regions like Lima managed the crisis more effectively. Furthermore, the resulting economic downturn severely impacted regional growth, especially in areas dependent on tourism and mining, which were affected by declining global demand and mobility restrictions [42].

### Post Pandemic effects (2022–2023)

Between 2022 and 2023, regions with strong economic foundations in mining, manufacturing, and exports, such as Lima, Arequipa, and La Libertad, showed signs of recovery, improving their competitiveness thanks to their infrastructure and connectivity. However, Amazonian regions such as Loreto and Ucayali continued to lag due to deficiencies in infrastructure and access to essential services. Lima led in innovation and digitalization, but the rest of the country, except for some southern cities, showed limited progress due to a lack of technological investment. Despite infrastructure improvements, environmental sustainability remains to be fully integrated in many regions. In addition, political instability and inequalities in education and health negatively affected regional competitiveness, with Lima and Callao maintaining the lead in the Regional Competitiveness Index [43].

In the Peruvian context, geographic and socioeconomic diversity presents unique challenges for measuring and comparing competitiveness across its regions. Traditionally, this measurement has been done using standard statistical methods that, while helpful, often fail to capture the complexity and dynamics of regional factors. This is where the application of non-linear machine learning (ML) models offers an innovative approach. Due to their ability to handle large volumes of data and learn complex patterns, these models can provide more profound and more accurate insights into regional competitiveness. Previous research has shown that ML has been effectively applied in diverse fields such as medicine, biology, and finance. However, its application in measuring regional competitiveness is still in its early stages. These studies have documented how non-linear models are well suited for data with complex and non-linear relationships, as is typically the case in regional studies where multiple variables interact unpredictably. However, despite these advances, there is a significant gap in the specific literature on applying these advanced ML models in measuring competitiveness in the Peruvian context. Most studies have focused on regions with more homogeneous economies and data structures, leaving aside regions with high diversity and inequality, such as those in Peru. This gap in knowledge underlines the need to explore how non-linear ML models can be adapted and applied to capture and analyze the complexity of regional competitiveness in such a varied context.

The overall objective of this research was to examine the effect of applying non-linear ML models in measuring the performance of the Peruvian Regional Competitiveness Index (PRCI) at the subnational level. To achieve this objective, a formative index was developed based on five critical dimensions: economy, government, infrastructure, businesses, and people, which were in turn subdivided into a total of 91 indicators or suitability indices. This multi-dimensional and granular approach allowed for a more precise and detailed assessment of regional competitiveness, providing a solid basis for applying ML models.

In conclusion, this research filled an essential gap in the existing literature and provided a robust and replicable methodology for assessing regional competitiveness in similar contexts. This study contributes significantly to Peru’s regional analysis and evidence-based policy development, highlighting the importance of integrating advanced technologies such as ML into territorial planning and management.

### Peru’s regional competitiveness index

Effectively measuring regional competitiveness in Peru represents a significant challenge due to the complex interaction of multiple socioeconomic variables and the country’s geographic diversity. Traditional measurement methods often fail to capture these complex relationships and the dynamics between the factors contributing to Peru’s Regional Competitiveness Index (RCI) [43]. This limitation becomes especially evident in a country with 25 regions presenting marked differences in economic development, infrastructure, human capital, and institutional capacity. Regional competitiveness is a multidimensional concept requiring advanced analytical approaches for correct measurement and understanding [44].

The central problem lies in the need to develop a more accurate and robust predictive model that can:

1. Efficiently process and analyze the 91 variables distributed across five fundamental pillars (economy, government, infrastructure, businesses, and people).
2. Capture the non-linear relationships between these indicators.
3. Provide reliable Peruvian Regional Competitiveness Index predictions that reflect the multidimensional reality of each region.

The conventional statistical methods have demonstrated limitations in handling this complexity, especially in contexts where relationships between variables are non-linear and subject to significant temporal changes, as evidenced during the COVID-19 pandemic (2020-2021) and the post-pandemic period (2022-2023) [45]. It is emphasized that this situation demands the implementation of machine learning techniques that can adapt to the dynamic nature of the data [46].

- Efficiently manage the interdependence between variables.
- Generate more precise predictions that consider the particularities of each region.

Solving this problem is crucial to facilitating informed decision-making at the public policy level [47]. This allows for a more efficient distribution of resources and identifies priority areas of intervention in each region [48]. Developing more effective strategies to reduce regional competitiveness gaps is also stressed [49].

Applying machine learning techniques to analyze regional competitiveness has shown promising results in various international contexts [50].

However, its implementation in emerging economies such as Peru presents unique challenges due to the data’s heterogeneity and the specific socioeconomic context characteristics [51].

Therefore, this study focuses on developing and evaluating non-linear machine learning models that can address these limitations and provide a more accurate tool for measuring and predicting the ICRP. This approach can significantly contribute to a better understanding and managing regional competitiveness in developing economies [52], providing more accurate insights for evidence-based public policy formulation [53].

## Methodology

### Objective of the study

The present research aims to evaluate the use of non-linear machine learning models in the measurement of Regional Competitiveness in the 25 regions of Peru, through the indicators contained in the 5 pillars: economy, government, infrastructure, companies and people.

### Data & study of period

The research covers 25 regions in Peru and focuses on the period 2016-2023. In each region, the annual score of these five pillars is analyzed, both at the level of the suitability index and the general competitiveness index achieved.

The data comes from the Research Center on Competitiveness, Corporate Finance, and Public Policies of CENTRUM PUCP, which collects data from official sources in Peru, such as Ministries and the National Institute of Statistics and Informatics (INEI).

Together with the Pontifical Catholic University of Peru (CENTRUM PUCP), these entities prepare the PRCI, identifying the factors necessary for each region’s sustainable development and analyzing its evolution since 2016.

The PRC Index is essential to understanding each region’s current situation, and its monitoring allows for the formulation of policies, the promotion of investments, and the elaboration of Regional Development Plans.

**Fig 2:**
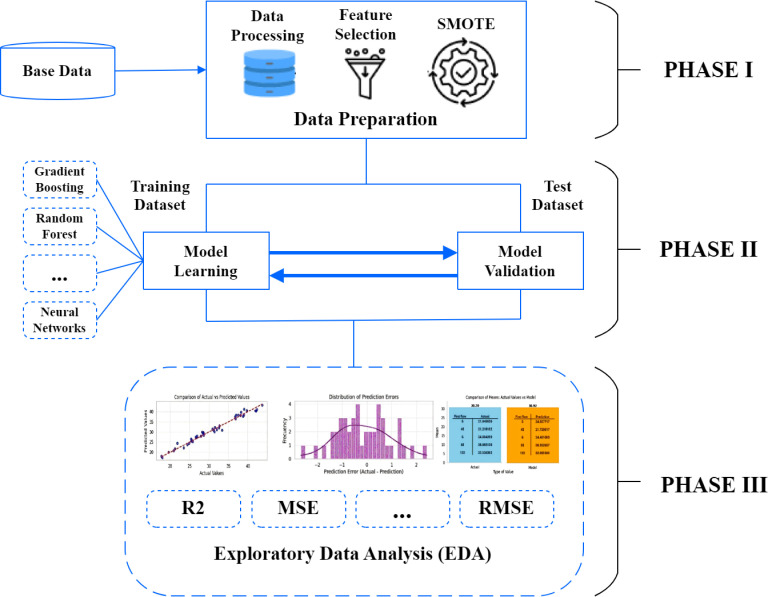
Overview of the proposed methodology.

The research is carried out in three steps (Fig 2).

**Fig 3:**
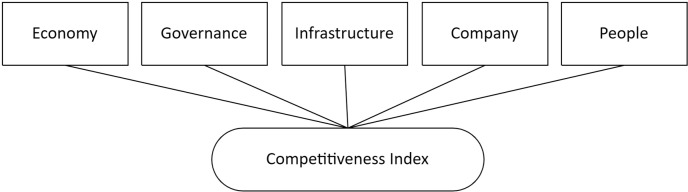
Five dimensions of competitiveness.

First, to prepare the data, we work with each of the 25 regions of the country through five pillars of competitiveness: economy, government, infrastructure, companies, and people (Fig 3). Using this information, a dataset (S1 File) was prepared, which included these variables and the corresponding PRC Index for the period 201-2023.

It should be noted that the selection of competitiveness pillars has been made based on the factors that would have a positive impact on the level of competitiveness of the regions, aligning them with National Objective No. 3, “Raise the levels of competitiveness and productivity with decent employment and based on the sustainable use of resources, human capital, the intensive use of science and technology, and the digital transformation of the country” of the National Development Strategic Plan to 2050, which is a strategic commitment to the development of the country in the medium and long term [36].

Second, we collect the dataset published from 2016 to 2023 and split it into a training set (70%) that estimates the model parameters and a test set (30%) that estimates the model accuracy.

Third, we performed an Exploratory Data Analysis (EDA) on the competitiveness pillars dataset to understand the structure and characteristics of the variables. This preliminary analysis allowed us to identify meaningful relationships between the pillars and other factors affecting regional competitiveness through techniques such as correlation analysis. We also calculated basic statistical measures, such as mean, median, and standard deviation, to examine the data distribution and detect potential outliers that could influence the analysis. This process provided a solid foundation for building predictive models and performing more detailed studies.

### Variables

First, using the reports collected from each region’s Peruvian Regional Competitiveness Index (PRCI), we used 91 indicators, also called suitability indexes, as explanatory variables. These indicators were grouped into the five pillars of regional competitiveness: economy, government, infrastructure, companies, and people [29]. These pillars will be used to evaluate the model’s predictive capacity.

The suitability index for the present study is listed in Table 1.

**Table 1:** Suitability index by each pillar.

Economy pillar		
Real Gross Domestic Product	External Insertion	Economically Active Employed Population
Actual Gross Domestic Product Per Capita	Volume of Final Exports	Relative Economically Active Employed Population
Real Gross Domestic Product Growth	Exporting dynamics	Average Independent Income
Real Gross Domestic Product Per Capita Growth	Destination Countries	Average Income Salaried Employer
Value of Final Exports	Export Products	
**Governance Pillar**		
Total Resources	Progress of Execution	Terrorism
Total Resources Per Capita	Executed Expenditure	Processing of Files
Fiscal autonomy	Crimes	Resolved Files
Collection Efficiency	Faults	
**Infrastructure Pillar**		
Electrical energy	Density of the Paved Neighborhood Road Network	Number of Hotels
Average Electricity Billing to Free Customers	Paved Neighborhood Road Network	Number of beds
Free Clients	Land Transport Density	Overnight stays of foreigners
Average Electricity Billing to Regulated Customers	Land Transport	Overnight Stays for Nationals
Regulated Clients	Air Transport Density	Other Establishments
Actual National Paved Road Density	Air transport	Fixed Telephony Density
National Paved Road Network	International Cargo Traffic at Airports	Fixed Telephony
Density of the Paved Departmental Road Network	Arrival of Guests	Cellular Telephony Density
Paved Departmental Road Network	Hosting Capabilities	Internet Services
**Companies Pillar**		
Average Labor Productivity	Presence of Successful Companies	Creation of New Products or Services
Economically Active Population Employed 14 Years and Olde	Managerial Capacity	Improvement of Techniques and Processes
Number of Companies	Long Term Vision	Access to well-paid positions
Penetration of the Financial System	Adaptability	Opportunity for Independents
Financial System Coverage	Internationalization capabilities	Stable Employment
Birth of Companies	Existence of Innovative Products/ Services	Salary Level
Effort to develop businesses	Cases of Innovative Companies or People	Over Labor Cost
**People Pillar**		
Primary Text Comprehension	Number of Formation Center of technology	Studies Achieved
Secondary Text Comprehension	Number of University students undergraduates, and professionalized	Illiteracy
Secondary Math Tests	Density of Technological Training Centers	Life expectancy
Density of University students undergraduates	Number of Technological Training Centers	Health Insurance Affiliation
Number of University students undergraduates	Density of Occupational Training Centers	Malnutrition
Density of Formation Center of technology	Number of Occupational Education Centers	

### Predictive models

The variables use predictive model regularization techniques to avoid overfitting and improve model generalization [37]. The predictive models used have specific regularization mechanisms to control overfitting, such as:

- Trees and Random Forest: Limits the depth and number of samples needed to create splits.
- Boosting (AdaBoost, XGBoosting, Gradient Boosting): Adjusts the number of estimators and the learning rate.
- Neural Networks: Uses techniques such as Dropout, L2 regularization, and Early Stopping.

Proper tuning of these parameters is critical for the model to generalize correctly without overfitting the training data [38]. Secondly, we selected six supervised predictive modeling techniques:

### Decision tree

A decision tree is a visual tool representing choices and their outcomes in a tree-like structure. Nodes in the graph depict events or decisions, while the edges represent decision rules or conditions. Each node corresponds to attributes within a group to be classified, and each branch represents a potential value for the node.

**Table:** 

**Algorithm 1: Decision Tree Pseudo Code**
Input: D:training set;F:set of available features;L:maximumnumberoflevels n:minimum impurity threshold Process Initialize the tree *T* as a root node. Create root node with data *D* and features *F* For each node *n*, perform the following steps: Evaluate node n: If *η* contains all samples of the same class or the impurity of *n* is less than the threshold *η*, label the node as leaf and stop splitting. If node level *n* is equal to *L*, label it as leaf and stop splitting. • Find the best split (f*,v*) for the node: For each characteristic *f* ∈ *F* For each value *v* of *f*: Divide *D* into two subsets, *D*_left_ and *D*_right_, such that: *D*_*Lefth*_ ={x ∈ D | *f*(x) ≤ v}, *D*_Rigth_ ={ x ∈ D | *f*(x) > v} Calculate the impurity (Gini, entropy, or variance) after partitioning. Select the split (f*, v*) that minimizes the impurity in the resulting nodes. Split node *n* into two child nodes: Create a child _*left*_ *η*_l*eth*_ for *D*_*left*_ Create a child _*right*_ *η*_*rigth*_ for *D*_*rigth*_ 4 Stop building the tree when all nodes have been classified, or no more valid splits are possible according to *η* and *L* Output: T:a trained decision tree

### Random Forest

Random Forest builds multiple decision trees trained with different samples (using Bootstrap) and with random feature selection at each node. Combining all the trees’ predictions through voting (in classification) or averaging (in regression), Random Forest improves accuracy and reduces overfitting compared to a single decision tree.

**Table:** 

**Algorithm 2: Random Forest Pseudo Code**
Input: *D* = training dataset *T*: number of trees *m*: number of features to select at each node split Process For *t = 1,…,T*: Generate data subset *Dt* using bootstrap. Training tree *ℎt* on *Dt*: For each node: Randomly select *m* features. Split the node using the best feature among the *m*. Repeat until the stopping criterion is met. Save the tree *ℎt*. For each sample *x*, make a prediction using each tree *ht*. In classification, use majority voting: $H\left(x\right)=mode\left(\left\{h_{1}\left(x\right),h_{2}\left(x\right),\ldots h_{T}\left(x\right)\right\}\right)$ In regression, use the average of the predictions: $H\left(x\right)=\frac{1}{T}\overset{T}{\underset{t=1}{\sum }}h_{t}\left(x\right)$ Output: *H(x)*: combined prediction of all trees.

### Extreme Gradient Boosting

Extreme Gradient Boosting is a machine learning algorithm that builds decision trees sequentially to optimize accuracy. It minimizes a loss function using gradient descent, adjusting each tree based on prior errors. XGBoost includes regularization, pruning, and learning rate adjustments to prevent overfitting and improve generalization. Known for its efficiency, it handles large datasets with fast training. XGBoost is widely used for classification, regression, and ranking tasks.

**Table:** 

**Algorithm 3: XGBoost Pseudo Code**
Input: $D:\text{training set};L:numberofiterations\left(numberoftreestobuild\right)$ η:learning rate;λ:L2regularization;γ:L1regularization $\Omega \left(h\right):\text{tree regularization term}$ *Objective*: loss function *L(θ)*, typically based on the squared error or *log-loss* Process Initialize predictions: Initialize the model with a constant initial prediction for all data points: ${\hat{y}}_{0}=\frac{1}{n}\overset{n}{\underset{i=1}{\sum }}yi$ For each iteration *t* = 1 until *L*: Calculate *gi* and *hi* (gradient and Hessian) for each *i* = 1,…, *n*. $gi=\frac{\partial L\left(yi,\hat{y}i^{\left(t-1\right)}\right)}{\partial \hat{y}i^{\left(t-1\right)}}$ $hi=\frac{\partial^{2}L\left(yi,\hat{y}i^{\left(t-1\right)}\right)}{\partial {\left(\hat{y}i^{\left(t-1\right)}\right)}^{2}}$ b Building the tree *ht*: $\text{Ganancia}\left(f,v\right)=\frac{1}{2}\left(\frac{{\left(\sum_{i\in izq}g\right)}^{2}}{\sum_{i\in izq}hi+\lambda }+\frac{{\left(\sum_{i\in der}g\right)}^{2}}{\sum_{i\in der}hi+\lambda }-\frac{{\left(\sum_{i}g\right)}^{2}}{\sum_{i}hi+\lambda }\right)-\gamma$ c Tree pruning: If the gain in the objective function does not exceed a threshold, the split is removed to avoid overfitting. d Update predictions: $\hat{y}i^{t}=\hat{y}i^{\left(t-1\right)}+\eta ht\left(xi\right)$ Regularization: Apply regularization to control model complexity: $\text{Ω}\left(h\right)=\gamma T+\frac{1}{2}\lambda \overset{L}{\underset{j=1}{\sum }}w_{j}^{2}$ Where *T* is the number of terminal nodes (leaves), and *wj* are the weights of the leaves Repeat steps 2 to 6 until you have completed L iterations (trees). Return the final model: The final model is a weighted combination of all the trees built $\hat{y}=\overset{L}{\underset{t=1}{\sum }}\eta ht\left(xi\right)$ Output: Model *H*: an ensemble of sequentially fine-tuned decision trees

### Adaptive Boosting

The AdaBoost algorithm builds an ensemble of sequentially trained weak classifiers. At each iteration, it adjusts the weights of the samples, giving higher weight to the misclassified samples. The weak classifiers are weighted according to their accuracy, and the final prediction combines all of them through weighted voting.

**Table:** 

**Algorithm 4: AdaBoost Pseudo Code**
Input: *D* = $\text{\{}(x_{1},y_{1}\text{)},\ldots \ldots \left(x_{n},y_{n}\right)\}$ : training dataset with features *xi* and labels *yi* *T*: number of iterations or models (estimator). $h_{t}\left(x\right):$ weak classifier (usually a small decision tree, or stump). $w_{i}:$ weights assigned to each sample at each iteration. Process: Initialize $t=1,\ldots ,T:\;w_{i}^{\left(1\right)}=\frac{1}{n},\forall i$ To *t* = 1, ..., *T*: Train classifier *ht*(*x*) using the weights $w_{i}^{\left(t\right)}$ Calculate the error ∈ *t* of *ht*(*x*): $\in_{t}=\overset{n}{\underset{i=1}{\sum }}wi^{\left(t\right)}.1\left(h_{t}\left(x_{i}\right)\neq y_{i}\right)$ Calculate $\alpha_{t}=\frac{1}{2}\text{log}\left(\frac{1-\in t}{\in t}\right)$ Update the weights $w_{i}^{\left(t+1\right)}$: $w_{i}^{\left(t+1\right)}=w_{i}^{t}.\text{exp}\left(\propto_{t}.1\left(h_{t}\left(x_{i}\right)\neq y_{i}\right)\right)$ Normalize $w_{i}^{\left(t+1\right)}$ so that they add up to 1. Return $H_{x}=sign(\sum_{t=1}^{T}\propto_{t}h_{t}\left(x\right)$ Output: Return the final classifier (*x*), a weighted combination of the trained weak classifiers.

### Neural Networks

Neural Networks are models organized in layers (input, hidden, output) that capture complex non-linear relationships by adjusting weights and activation functions. They are trained using backpropagation, where errors are propagated backward to update weights and biases. This process uses gradient descent to minimize the loss function and improve accuracy. Neural networks are highly flexible and powerful, capable of learning complex patterns.

**Table:** 

**Algorithm 5: Neural Networks Pseudo Code**
Input: *D*: training dataset. *L*: number of layers. *Nl*: number of neurons in each layer. *η*: learning rate. *f*: activation function. (*y*, ŷ): loss function. Process Initialize the weights *W*_*l*_ and bias *b*_*l*_ randomly. For each epoch *t* = 1,…,: For each sample (*xi*,*yi*) ∈ *D*: Forward Propagation: Compute ŷ_*i*_ using *H*(*x*). Calculate the loss *E*(*yi*, ŷ*i*) Backpropagation: Compute the gradients of the loss concerning *W*_*l*_ and *b*_*l*_ Update weights and bias: $W_{l}=W_{l}-\eta .\frac{\partial \text{E}}{\partial W_{l}}$ $b_{l}=b_{l}-\eta .\frac{\partial \text{E}}{\partial b_{l}}$ Return (*x*). Output: *H* (x): trained neural network.

### Gradient Boosting

Gradient Boosting is a robust ensemble learning algorithm that combines multiple weak decision trees sequentially to create a strong predictive model. It works by iteratively fitting new trees to the residual errors of previous predictions, using gradient descent to minimize a loss function. Each new tree focuses on correcting the ensemble’s mistakes, while a learning rate prevents overfitting.

**Table:** 

**Algorithm 6: Gradient Boosting Pseudo Code**
Input: *D:* training dataset: {(x_1_,y_1_),..., (x_n_,y_n_)} *L(y,F(x)):* loss function *M:* number of iterations *η:*learning rate Process Model Initialization: *F*_*0*_*(x)* = *argmin_γ* ∑* L(y*_*i*_*,γ)* For m = 1 to M: a Residual Calculate: r_i__m_ = *-[∂L(y*_*i*_*,F(x*_*i*_*))/ ∂ F(x*_*i*_*)]_{F = F*_*m-1*_*}* For each observation *i = 1,...,n* b Fit regression tree: h_m_(x) = fit_decision_tree(x, r) Fitting a decision tree to the residuals Define the terminal regions *R*_*jm*_, *j = 1*,...,*J*_*m*_ c Calculate the values of the leaves: *γ*_*jm*_ *= argmin_γ* ∑* _{x*_*i*_ ∈ *R*_*jm*_*} L(y*_*i*_*,F*_*m-1*_*(x*_*i*_*)* +* γ)* For each region *j = 1,...,J*_*m*_ d Update the model: *F*_*m*_*(x) = F*_*m-1*_*(x) + η* * ∑_*r*_ *γ*_*jm*_ *I(x* ∈* R*_*jm*_*)* Complexity control: Maximum tree depth Minimum number of observations per node Tree pruning based on cross-validation Regularization: Apply shrinkage (η < 1) to control overfitting Random subsampling of data (stochastic gradient boosting) 5 Repeat steps 2-4 until M iterations are complete Output: Final model *F_M(x):* weighted sum of decision trees

## Analysis and results

In this section, we provide the results found by the research models: Decision Tree, Random Forest, Extreme Gradient Boosting, Adaptive Boosting, Neural Network, and Gradient Boosting.

**Fig 4:**
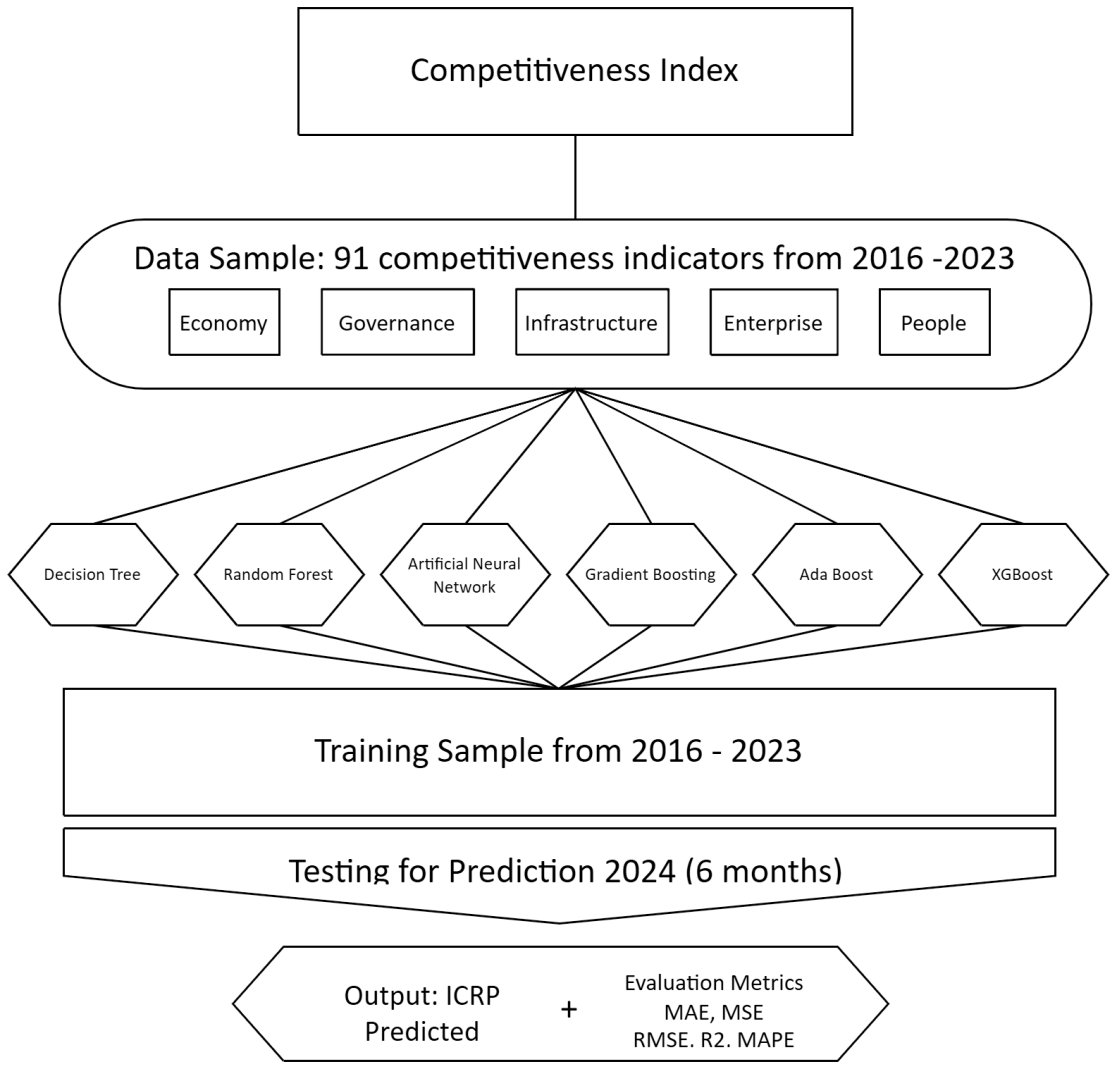
Graphical summary. 91 variables suggested by the Research Center on Competitiveness, Corporate Finance, and Public Policies of CENTRUM PUCP were selected. Machine learning models used: Decision Tree, Random Forest, Artificial Neural Networks, Gradient Boosting, Ada Boost, XGBoost. Results are presented as Predicted PRCI and Evaluation Metrics.

We initially compare and contrast the results based on observed historical values of the previously trained models; subsequently, we test the models again in the framework of multiple periods to validate them. Fig 4 illustrates our methodology.

### Comparative analysis

Six machine learning (ML) models were evaluated, as shown in Tables 2 and 3, with the comparative analysis of the machine learning models revealing a distinct hierarchy in their predictive performance. The Gradient Boosting model stands out with the lowest Mean Absolute Error (MAE = 0.8746) and a higher Mean Absolute Percentage Error (MAPE = 2.8935%), closely followed by the XGBoost, which exhibits a slightly higher Coefficient of Determination (R^2^ = 0.9729) and consistently low error metrics (MAE = 0.9792, MSE = 1.5305).

**Table 2:** Evaluation metrics of XGBoost, AdaBoost, and gradient boosting models.

Evaluation Metrics	XGBoost	AdaBoost	Gradient Boosting
**Mean Absolute Error (MAE)**	0.9792	1.0486	0.8746
**Mean Square Error (MSE)**	1.5305	1.7937	1.1399
**Root Mean Square Error (RMSE)**	1.2371	1.3393	1.0677
**Coefficient of Determination (R** ^2^ **)**	0.9729	0.9635	0.9756
**Mean Absolute Percentage Error (MAPE)**	3.3442%	3.6949%	2.8935%

**Table 3:** Evaluation metrics of simple decision tree (TREE), random forest, and Artificial Neural Network Model (ANN).

Evaluation metrics	TREE	Random forest	ANN
**Mean Absolute Error (MAE)**	1.3347	0.9681	1.2274
**Mean Square Error (MSE)**	2.9633	1.3469	2.7821
**Root Mean Square Error (RMSE)**	1.7214	1.1606	1.6680
**Coefficient of Determination (R** ^**2**^ **)**	0.9397	0.9726	0.9507
**Mean Absolute Percentage Error (MAPE)**	4.7090%	3.2274%	4.0942%

The Random Forest emerges as the third most effective model with robust performance (MAE = 0.9681, R^2^ = 0.9726), outperforming the AdaBoost, which shows intermediate results (MAE = 1.0486, R^2^ = 0.9635). In contrast, the simple decision tree (TREE) and the Artificial Neural Network exhibit significant limitations in their predictive ability, evidenced by their higher errors (TREE: MAE = 1.3347, ANN: MSE = 2.7821). This comprehensive metrics evaluation suggests that Gradient Boosting provides the most accurate modeling for this specific dataset. However, the difference with XGBoost is marginal, thus establishing that both models are highly competent for this particular application, with a slight advantage for Gradient Boosting in terms of absolute and percentage error.

**Fig 5:**
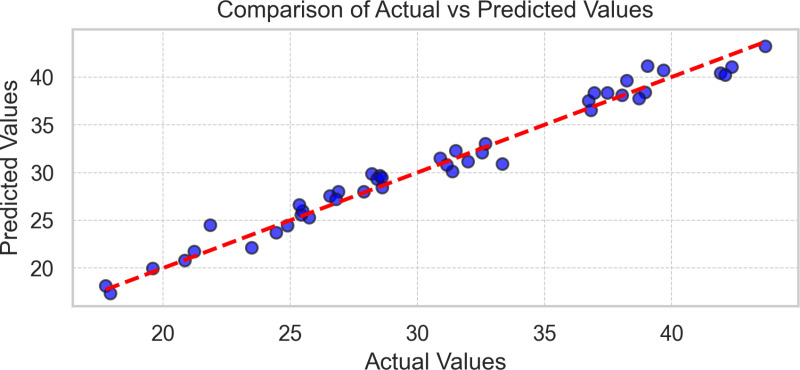
Scatterplot of Gradient Boosting Model where comparison of actual vs predicted values.

The scatter plot comparing actual versus predicted values of the Gradient Boosting model where the red dotted line represents the perfect prediction and the blue dots are the individual predictions, where it reveals a strong linear correlation with a high coefficient of determination 0.9756, maintaining accuracy for both low and high values, with uniform prediction intervals. An excellent model performance is observed, with no significant biases or outliers (Fig 5)

**Fig 6:**
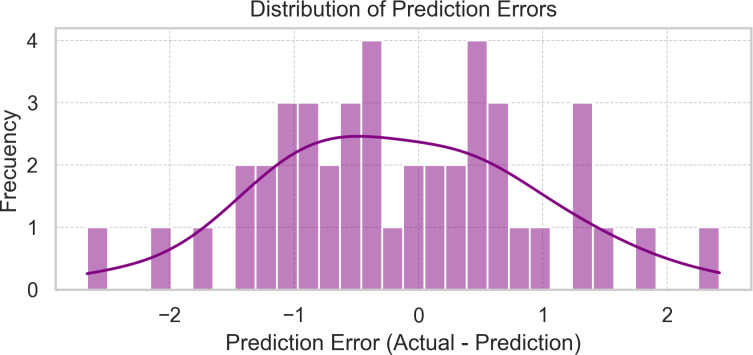
Histogram of prediction error distribution for the Gradient Boosting Model.

The histogram of the prediction error distribution for the Gradient Booster model reveals an approximately regular pattern (Fig 6). The superimposed density curve (purple line) confirms the approximation to a normal distribution, albeit with slight asymmetries.

This distribution suggests that the Gradient Boosting model makes generally accurate predictions, with most errors concentrated near zero and only a few cases presenting more significant deviations at the extremes. This supports the model’s effectiveness as evidenced by its evaluation metrics, particularly its low MAE of 0.8746 and MAPE of 2.8935%.

**Fig 7:**
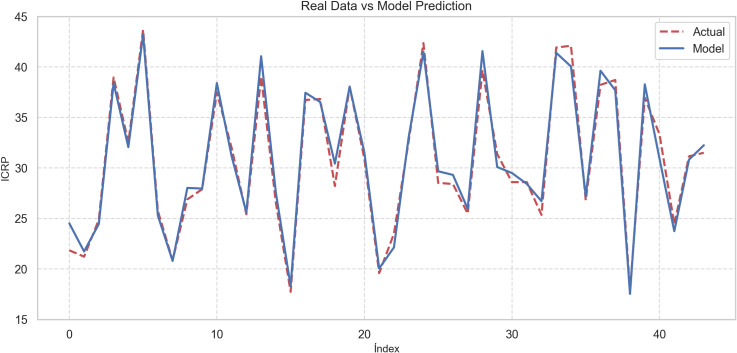
Comparison of actual data vs Gradient Boosting Model prediction.

A graph comparing the actual values (dotted red line) and the predictions of the Gradient Boosting model (solid blue line) for the Peruvian Regional Competitiveness Index (PRCI) over different observations. The model follows the trend of the actual data very closely. The predictions show high accuracy, demonstrating the ability of Gradient Boosting to model the variability of the regional competitiveness index adequately (see Fig 7).

**Fig 8:**
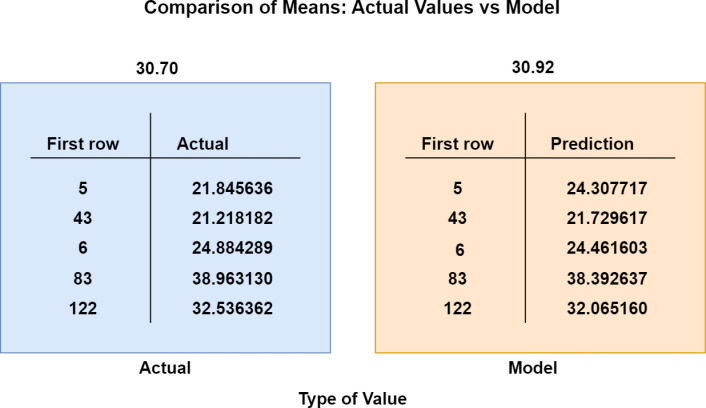
Comparison of means of actual values vs values model.

Finally, presents a bar chart that clearly illustrates the differences between the actual and modeled values, enabling a quick visual assessment of the model’s accuracy in terms of the mean of its predictions (Fig 8).

The results show that the mean of the actual values is 30.70, while the model’s mean is 30.92, resulting in a difference of 0.22 (0.71%). This comparison of means provides insights into any systematic bias in the model’s predictions. If the model’s mean differs significantly from the actual mean, it may indicate a tendency to overestimate or underestimate values. A model mean close to the actual mean suggests that, on average, the model’s predictions are well-centered around the exact values.

### Results

The result is that in the Gradient Boosting model, on average, the predictions deviate 1.0411 units from the actual value in the calculated MAE; for the RMSE, the standard deviation of the prediction errors is 1.5541 units and penalizes significant errors more than the MAE. The R^2^ of the model explains 97.34% of the variability in the data and achieves an excellent fit; for the MAPE found on average, the predictions have an error of 3.2780%, with the mistake deficient concerning the other machine learning models used in this research.

**Fig 9:**
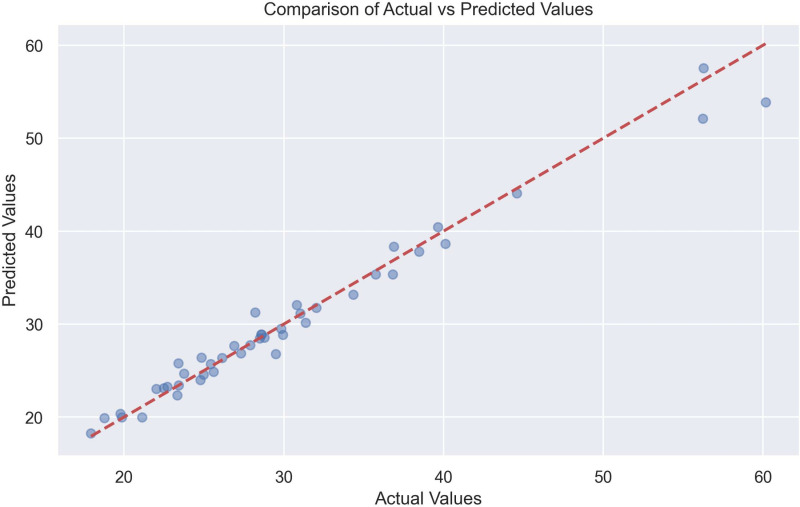
Scatter diagram of the results where the actual values are compared with the predicted ones.

A scatter plot comparing actual and predicted values of the Regional Competitiveness Index using the Gradient Boosting model. It reveals a strong positive linear correlation of 20 to 60 points (Fig 9).

The absence of systematic patterns of deviation and outliers validates the robustness of the model. At the same time, the concentration of points close to the diagonal line demonstrates consistent accuracy at various levels of the competitiveness index. The results confirm that the Gradient Boosting model efficiently captures the variability of data from any region of the country evaluated, generating reliable estimates for decision-making.

## Conclusions

Machine learning techniques can help quantify the competitive performance of the 25 regions in multiple pillars identified as key for competitiveness by policymakers. Let’s consider regional competitiveness as an unobservable variable that regions try to maximize. Machine learning models offer a method to rank the success of areas in this task without imposing strict assumptions on the function that transforms these pillars into competitiveness levels. The proposed method only requires assuming that regions are comparable at some level (an essential requirement to obtain a meaningful ranking) and that their behavior reflects a certain level of optimality in the indices obtained for each region in the best possible way.

While the use of machine learning techniques to rank multiple pillars is not new, we expand their applicability by using non-linear models to project the behavior of the competitiveness index in the coming years. This allows us to analyze the evolution of competitiveness in the country. In the case of Peru, the proposed model suggests that the competitiveness index should gradually improve by 2024.

The study analyzes key factors for regional competitiveness such as the economy, governance, infrastructure, companies and people. However, it may not be sufficient to fully understand regional competitiveness. To get a more complete picture of the competitiveness of regions, future research can include factors such as the environment or the use/adoption of new disruptive technologies.

Another important limitation of the research is that it was conducted in a single country. Furthermore, the number of factors which constitute the pillars of regional competitiveness implies that replicating the study in other countries would require substantial efforts with regard to data collection and standarization. The methodology presented could be adapted and validated in other regions of Latin America and/or countries with different socioeconomic characteristics, evaluating its capacity to generalize the model to different contexts.

Analysing how each pillar affects competitiveness in a region would be valuable, despite the fact that non-linear models are highly accurate.The application of novel explainability methods, such as SHapley Additive Explanations (SHAP) and Local Interpretable Explanations of Independent Models (LIME), would provide significant insights. Specifically, the Gradient Boosting model could be effectively integrated with these highly accurate models to enhance the outcomes. In order to ascertain the rationale behind a model’s determination of a region’s competitiveness, LIME can be instrumental in generating a basic linear model directly derived from the region’s data. This approach facilitates a comprehensive understanding of the contributions of various elements, such as the economy and infrastructure, to the decision-making process. Alternatively, if the focus is on identifying areas with potential for achieving high levels of competitiveness, the random forest model is recommended, with SHAP being the optimal choice for investigating the characteristics that contribute to a region’s capacity to attain optimal results.

In addition, incorporating discourse with experts, rigorous examination of factual cases, and meticulous mathematical calculation techniques can significantly enhance the model’s reliability. This tests new and diverse options and makes the numbers in the model more meaningful. In addition, these discussions can be tested for legitimacy by using structural equations such as CB-SEM and PLS-SEM, which have been very useful in other qualitative research on competitiveness studies. It should be noted that combining quantitative and qualitative methods helps uncover new understandings of this research and provide better answers.

In summary, while this study demonstrates the utility of machine learning (ML) in addressing the complexity of Peru’s regional competitiveness data, its implementation can be viewed as one component within a broader, multidimensional approach. The findings presented can serve as a basis for future research that expands the understanding and improvement of the Competitiveness Index and other additional contexts that can be assessed and applied to similar countries worldwide.

## Supporting information

S1 File Database with the information used in the analysis. The database contains the indicators employed in the research and the ICRP for the years 2016–2023.
(XLSX)

## References

[1] Alexa D, Cismaș L, Rus A, Pop-Silaghi M. Economic growth, competitiveness and convergence in the European Regions. a spatial model estimation. Econ Comput Econ Cybern Stud Res. 2019;53:107–24. doi: 10.24818/18423264/53.1.19.07

[2] Kordalska AK, Olczyk M. Global competitiveness and economic growth: a one-way or two-way relationship? Equilibrium. 2016;11(1):121. doi: 10.12775/equil.2016.006

[3] Ferrarini F, Muzzioli S, De Baets B. A TOPSIS analysis of regional competitiveness at European level. Competitiv Rev. 2024;34:52–72. doi: 10.1108/CR-01-2024-0005

[4] Gabryelczyk R. Has COVID-19 accelerated digital transformation? Initial lessons learned for public administrations. Inf Syst Manage. 2020;37(4):303–9. doi: 10.1080/10580530.2020.1820633

[5] Kutnjak A. Covid-19 accelerates digital transformation in industries: challenges, issues, barriers and problems in transformation. IEEE Access. 2021;9:79373–88. doi: 10.1109/access.2021.3084801

[6] Marek K, Wińska E, Dąbrowski W. The state of agile software development teams during the Covid-19 pandemic. 2021;24–39. doi: 10.1007/978-3-030-67084-9_2

[7] Romero Bravo GJ, Espinoza Maza JDJ, Macgluf Issasi A, Suárez Álvarez A, Rodríguez Rodríguez LA. Aplicación de Machine Learning en la Indutria 4.0 en tiempos de pandemia. Interconectando Saberes. 2021;(11): doi: 10.25009/is.v0i11.2692

[8] Xu D, Xiao X. Retracted: influence of the development of VR technology on enterprise human resource management in the era of artificial intelligence. IEEE Access. 2025;1–1. doi: 10.1109/ACCESS.2020.3020622

[9] Sumets A, Kniaz S, Heorhiadi N, Farat O, Skrynkovskyy R, Martyniuk V. Methodical approach to selecting options for ensuring the competitiveness of enterprises in the system of development of agricultural clusters. Agric Res Econ. 2021;7:192–210.

[10] Annoni P, Dijkstra L, Gargano N. EU regional competitiveness index 2010. 2010.

[11] Bhawsar P, Chattopadhyay U. Competitiveness: review, reflections and directions. Global Bus Rev. 2015;16(4):665–79. doi: 10.1177/0972150915581115

[12] González Catalán SA. Regional competitiveness in Latin America: a comparative study of the key elements for regional performance. Invest Reg. 2021;50:125–46. doi: 10.38191/iirr-jorr.21.014

[13] Chrobocińska K. Comparative analysis of regional competitiveness in Poland from 2010–2019 in the context of the concept of sustainable development. Sustainability. 2021;13(6):3202. doi: 10.3390/su13063202

[14] Sánchez de la Vega JC, Buendía Azorín JD, Calvo-Flores Segura A, Esteban Yago M. A new measure of regional competitiveness. Applied Economic Analysis. 2019;27(80):108–26. doi: 10.1108/aea-07-2019-0010

[15] Veshneva I, Chernyshova G, Bolshakov A. Regional competitiveness research based on digital models using Kolmogorov-Chapman equations. 2021;141–54. doi: 10.1007/978-3-030-63563-3_12

[16] Law J. A dictionary of business and management. Oxford: Oxford University Press; 2016.

[17] Aiginger K. Competitiveness: from a dangerous obsession to a welfare creating ability with positive externalities. J Ind Compet Trade. 2006;6(2):161–77. doi: 10.1007/s10842-006-9475-6

[18] Ghicajanu M. Competitive analysis of the business with the Michael Porter model. Ann Univ Petrosani Economics. 2021;1:169–78. Available from: https://www.upet.ro/annals/economics/pdf/2021/18).%20Ghicajanu_2.pdf

[19] Díaz D, Alvarez B, Ojeda M. Competitividad regional y desarrollo económico Una breve Revisión de la literatura ecónomica moderna. Rev Economía Polít Buenos Aires. 2020;14:109–53. Available from: https://ojs.econ.uba.ar/index.php/REPBA/article/download/1720/2439?inl

[20] OECD. OECD regions at a glance 2011. OECD Publishing; 2011.

[21] Rădoi M, Șerban R. Regional innovation—a pillar of regional competitiveness and an object of regional development policy. J Adv Res Manag. 2019;10:35–43.

[22] Sleuwaegen L, Ramboer S. Regional competitiveness and high growth firms in the EU: the creativity premium. Appl Econ. 2020;52(22):2325–38. doi: 10.1080/00036846.2019.1686454

[23] Kitson M, Martin R, Tyler P. Regional competitiveness: an elusive yet key concept? Reg Stud. 2004;38(9):991–9. doi: 10.1080/0034340042000320816

[24] Bilbao-Terol A, Arenas-Parra M, Onopko-Onopko V. Measuring regional sustainable competitiveness: a multi-criteria approach. Operat Res. 2019;19(3):637–60. doi: 10.1007/s12351-017-0367-9

[25] Huggins R, Izushi H, Prokop D, Thompson P. Regional competitiveness, economic growth and stages. Proc Rijeka Facul Econ. 2014;32:255–83. Available from: https://www.efri.uniri.hr/upload/Nastavnici%20i%20istrazivanja/Arhiva%20Zbornika%20radova/10-huggins-izushi-prokop-thompson-2014-2-1420787458.pdf

[26] Januškaitė V, Užienė L. Intellectual capital as a factor of sustainable regional competitiveness. Sustainability. 2018;10(12):4848. doi: 10.3390/su10124848

[27] Möbius P, Althammer W. Sustainable competitiveness: a spatial econometric analysis of European regions. J Environ Planning Manage. 2020;63(3):453–80. doi: 10.1080/09640568.2019.1593005

[28] Sági J, Engelberth I. Regional development and well- being of regions in hungary. Polgári Szemle. 2018;14(Special Issue):184–94. doi: 10.24307/psz.2018.0412

[29] Carpio L Del, Feldman PM, Avolio B. Measuring regional competitiveness. Global Bus Rev. 2023; doi: 10.1177/09721509221145445

[30] International Monetary Fund. Peru: Selected Issues. IMF Staff Country Reports. 2001;01:1. doi: 10.5089/9781451831009.002

[31] Fishwick A. Labour Control and developmental state theory: a new perspective on import‐substitution industrialization in Latin America. Dev Change. 2019;50(3):655–78. doi: 10.1111/dech.12407

[32] OECD. Handbook on constructing composite indicators. Organisation for Economic Co-operation and Development; 2008.

[33] Schwab K. The global competitiveness report 2018. 2018. Available from: https://www3.weforum.org/docs/GCR2018/05FullReport/TheGlobalCompetitivenessReport2018.pdf

[34] Porter M. Competitive advantage of nations. Free Press; 1990.

[35] Benzaquen J, Del Carpio L, Zegarra L, Valdivia C. A competitiveness Index for the regions of a country. CEPAL Rev. 2010;67–84. Available from: https://repositorio.cepal.org/entities/publication/b17642b4-c51b-4e6b-962e-8b5c1a30c767

[36] Bronisz U, Heijman W, Miszczuk A. Regional competitiveness in Poland: Creating an index. Jahr Regionalwissen. 2008;28(2):133–43. doi: 10.1007/s10037-008-0026-y

[37] Dijkstra L, Annoni P, Kozovska K. A new regional competitiveness index: theory, methods, and findings. 2011.

[38] Iarossi G. Measuring competitiveness at the subnational level: The case of 37 Nigerian states. J Cent Cathedra. 2013;6(2):193–218. doi: 10.7835/jcc-berj-2013-0088

[39] Huggins R, Izushi H, Thompson P. Regional competitiveness: theories and methodologies for empirical analysis. J Cent Cathedra. 2013;6(2):155–72. doi: 10.7835/jcc-berj-2013-0086

[40] Amann E, Figueiredo P. Innovation, competitiveness, and development in Latin America. Oxford University Press; 2024.

[41] CENTRUM. Indice de competitividad regional del Peru 2019. 2019.

[42] CENTRUM. Indice de competitividad regional del Peru 2021. 2021.

[43] CENTRUM. Indice de competitividad regional del Peru 2023. 2023.

[44] Stanickova M, Melecký L. Understanding of resilience in the context of regional development using composite index approach: the case of European Union NUTS-2 regions. Reg Stud Reg Sci. 2018;5(1):231–54. doi: 10.1080/21681376.2018.1470939

[45] Madakkatel I, Zhou A, McDonnell MD, Hyppönen E. Combining machine learning and conventional statistical approaches for risk factor discovery in a large cohort study. Sci Rep. 2021;11(1):22997. doi: 10.1038/s41598-021-02476-9 34837000 PMC8626442

[46] Guryanova L, Milevskyi S, Piskun E, Belyaeva M, Kasyanenko L. Methods and models of machine learning in managing the competitiveness of audit companies. In: Guryanova L, Yatsenko R, Babenko V, Dubrovina N, editors. Machine Learning Methods and Models, Predictive Analytics and Applications Proceedings of the Workshop on the XII International Scientific Practical Conference “Modern problems of social and economic systems modelling” (MPSESM-W 2020). Kharkiv; 2020. 77 p.

[47] Androutsopoulou A, Charalabidis Y. A framework for evidence based policy making combining big data, dynamic modelling and machine intelligence. Proceedings of the 11th International Conference on Theory and Practice of Electronic Governance. New York (NY): ACM; 2018. pp. 575–583.

[48] Höchtl J, Parycek P, Schöllhammer R. Big data in the policy cycle: policy decision making in the digital era. J Org Comput Electron Commer. 2016;26(1-2):147–69. doi: 10.1080/10919392.2015.1125187

[49] Kouskoura A, Kalliontzi E, Skalkos D, Bakouros I. Assessing the key factors measuring regional competitiveness. Sustainability. 2024;16(6):2574. doi: 10.3390/su16062574

[50] Xu X, Chen Z, Chen S. Enhancing economic competitiveness analysis through machine learning: exploring complex urban features. PLoS One. 2023;18(11):e0293303. doi: 10.1371/journal.pone.0293303 37934756 PMC10629647

[51] Charles V, Zegarra LF. Measuring regional competitiveness through data envelopment analysis: a Peruvian case. Expert Syst Appl. 2014;41(11):5371–81. doi: 10.1016/j.eswa.2014.03.003

[52] Lei J. Research on the improvement path of international competitiveness of China’s agricultural product supply chain from the perspective of machine learning. Expert Syst. 2024;41(5). doi: 10.1111/exsy.12935

[53] Tacchella A, Zaccaria A, Miccheli M, Pietronero L. Relatedness in the era of machine learning. Chaos Solitons Fractals. 2023;176:114071. doi: 10.1016/j.chaos.2023.114071

